# Extracellular vesicles: mechanisms and prospects in type 2 diabetes and its complications

**DOI:** 10.3389/fendo.2024.1521281

**Published:** 2025-03-17

**Authors:** Zijian Liu, Ruiyang Yin, Jiaxing Tian

**Affiliations:** ^1^ Department of Endocrinology, The Affiliated Hospital of Changchun University of Chinese Medicine, Changchun, Jilin, China; ^2^ Graduate College, Beijing University of Chinese Medicine, Beijing, China; ^3^ Institute of Metabolic Diseases, Guang’anmen Hospital, China Academy of Chinese Medical Sciences, Beijing, China

**Keywords:** extracellular vesicles, diabetes, treatment, diabetic complications, mechanisms

## Abstract

Extracellular vesicles (EVs) are small vesicles released by cells into the surrounding environment, carrying biomolecules such as proteins, miRNA, etc., involved in intercellular communication and regulation of biological processes. With the continuous increase in the prevalence of diabetes, research on the relationship between extracellular vesicles and diabetes has attracted widespread attention. In this article, we specifically focus on the metabolic abnormalities related to EVs and diabetes, including obesity, inflammation, insulin resistance, β-cell damage, etc. We aim to explore how extracellular vesicles participate in the occurrence and development of diabetic complications, comprehensively examining the interactions between extracellular vesicles and key aspects of diabetes, forming a comprehensive and profound research framework. This is expected to provide important clues and insights for deepening our understanding of the pathophysiological mechanisms of diabetes.

## 1 Introduction

Diabetes is a chronic metabolic disease characterized by persistently elevated glucose levels in the blood. The main types of diabetes include type 1 diabetes, type 2 diabetes, gestational diabetes, and specific types of diabetes (1, 2). Among these, type 2 diabetes mellitus (T2DM) has the greatest impact, accounting for approximately 96% of all diabetes cases (3, 4). Globally, type 2 diabetes continues to pose a serious threat to public health and economic development, with global healthcare expenditures expected to exceed $1.05 trillion by 2045 (4). Type 2 diabetes is primarily caused by insulin resistance and/or insufficient insulin secretion, with progressive apoptosis of pancreatic β-cells playing a key role (5). Furthermore, poor glycemic control in patients with type 2 diabetes can lead to complications in both large and small blood vessels (5–7), which can result in heart attacks, strokes, kidney failure, and blindness, significantly increasing disability and mortality rates in these patients (6, 8). The continuous rise in these non-communicable diseases not only impacts individual health but also poses profound challenges to global economic and social development.

At present, the complex pathogenesis of type 2 diabetes has not been fully understood, and its complexity makes it difficult for a single or combined drug therapy to completely cure or halt its progression (9). Current hypoglycemic drugs mainly focus on blood sugar control and improving insulin sensitivity, but they do not effectively address the comprehensive pathological mechanisms of diabetes, such as inflammation, oxidative stress, and β-cell apoptosis. Moreover, current antidiabetic drugs often have side effects, resistance, and limitations in reversing the outcomes of complications (9–13). Significant individual differences among diabetic patients make it difficult for existing treatments to achieve precise and personalized interventions, leading some patients to fail to achieve optimal outcomes with current therapies (9). Therefore, exploring new therapeutic approaches to more comprehensively regulate the multiple pathological mechanisms of diabetes is particularly necessary. In this context, extracellular vesicles (EVs) have gradually attracted the attention of researchers.

**Figure 1:**
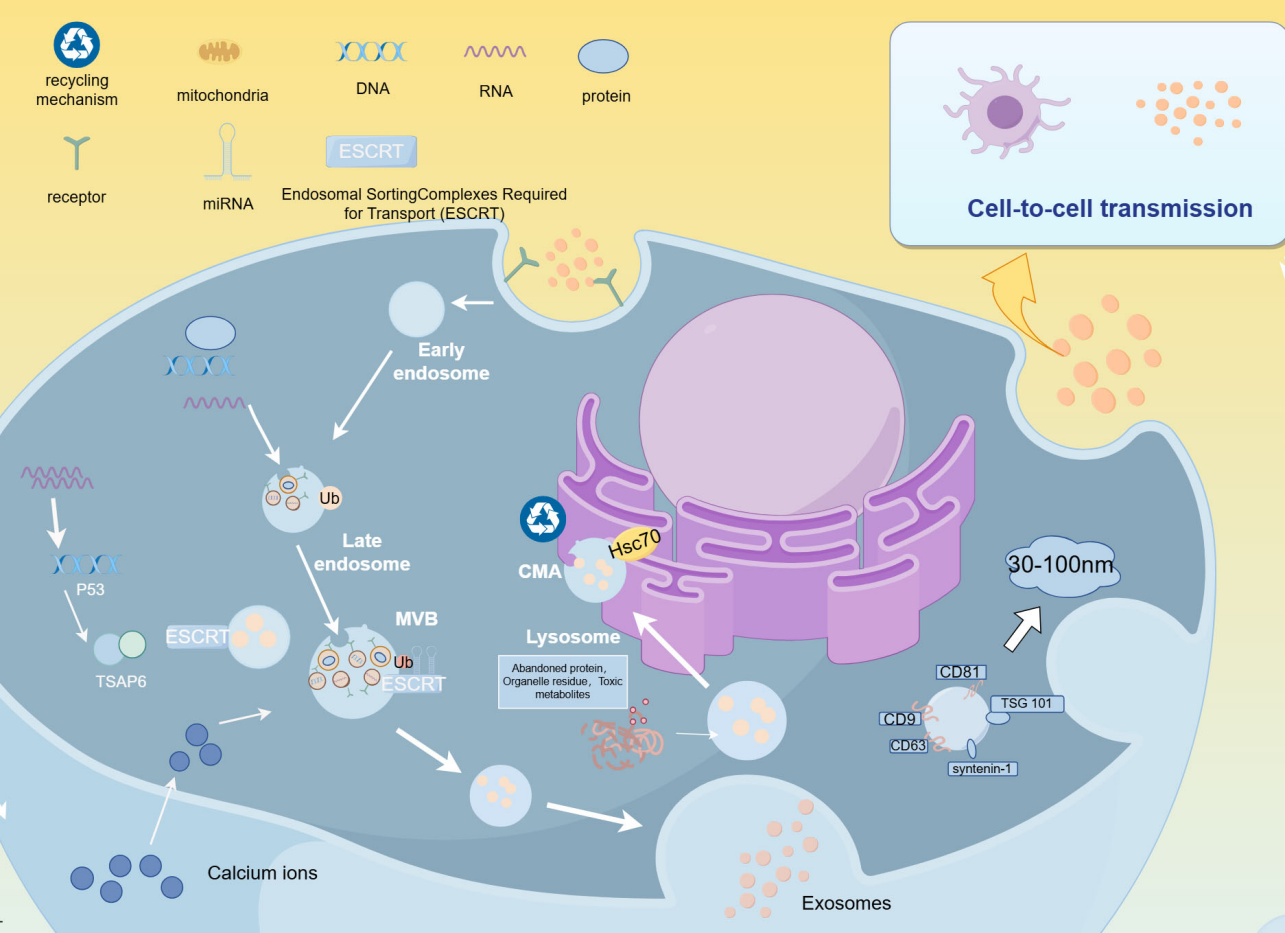
Biogenesis of EVs.

**Figure 2:**
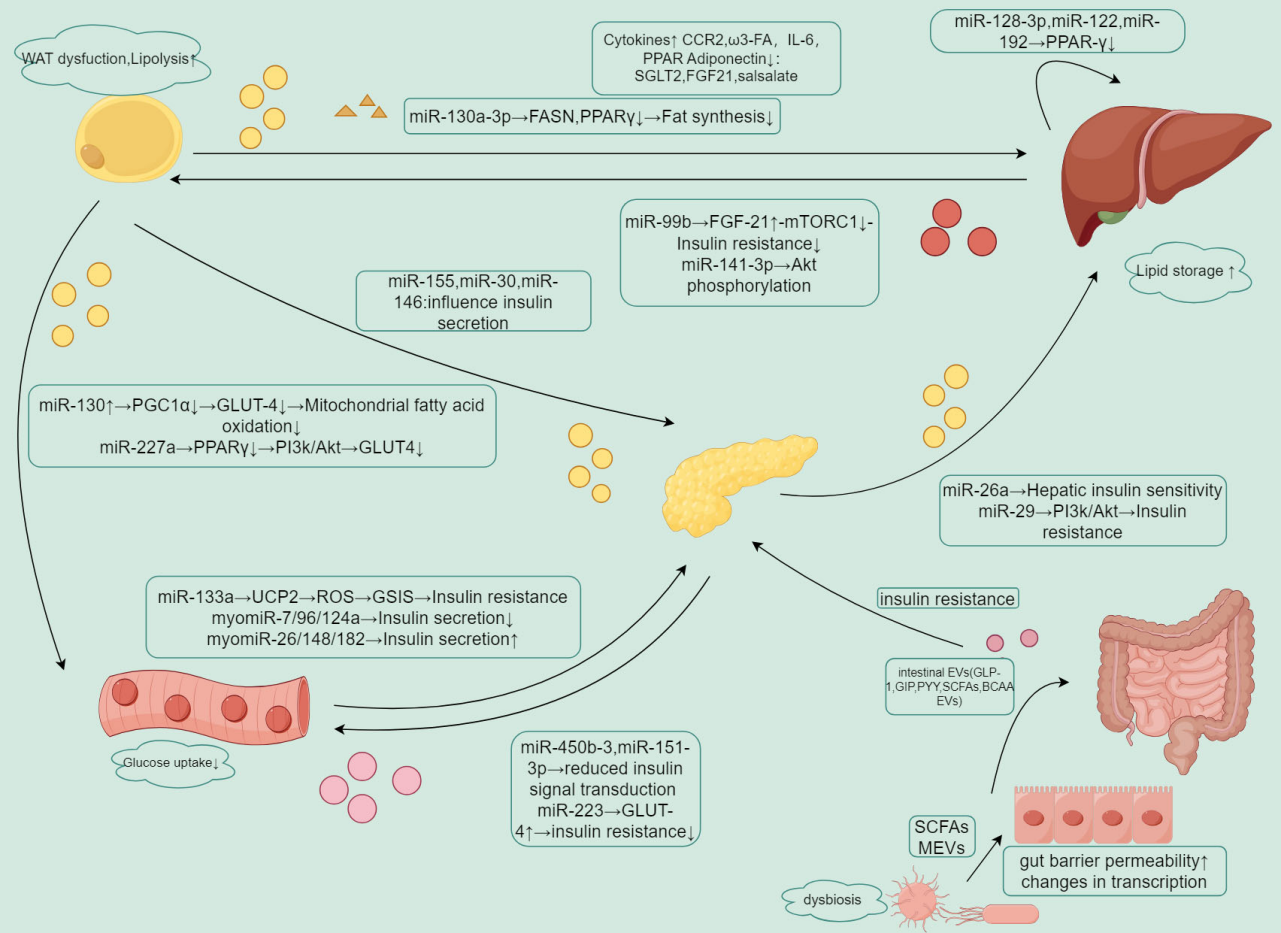
Summary of EV-mediated crosstalk between organs.

**Figure 3:**
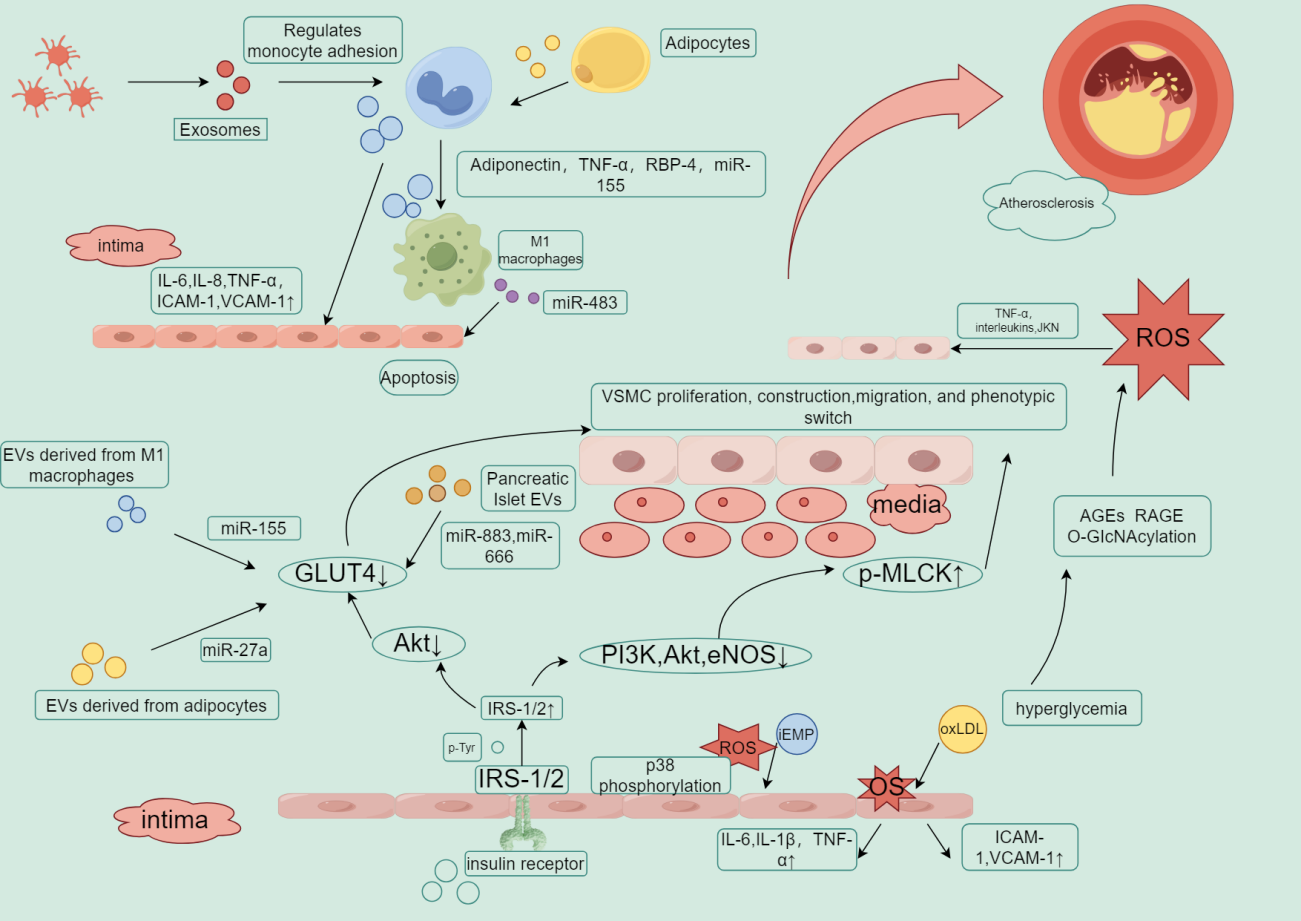
Key roles of EVs in insulin resistance, inflammation, and atherosclerosis.

Extracellular vesicles (EVs) are small membrane-bound vesicles released by cells into the extracellular environment, typically ranging in diameter from 30 nanometers to 1 micrometer. EVs mainly include exosomes (30–150 nm, released by multivesicular bodies), microvesicles (100–1000 nm, budding from the plasma membrane), and apoptotic bodies (500 nm to several micrometers, released by apoptotic cells) (14). As key mediators of intercellular communication, EVs can carry various bioactive molecules such as proteins, lipids, and miRNAs (14), exhibit strong targeted delivery capabilities, and are widely involved in intercellular communication and signal regulation. They directly participate in the regulation of core pathological mechanisms, such as cellular metabolism, inflammatory responses, and the protection of β-cell function (15). In **Figures 1**–**3** and **Table 1** of this article, we have summarized the mechanisms of EVs generation, the inter-organ crosstalk mediated by EVs, and the specific mechanisms by which the cargo of EVs induces diabetic vascular complications, aiming to explore the important roles of EVs in both physiological and pathological conditions. Given their unique multifunctionality and targeting capabilities, EV therapies have shown great potential in the treatment of diabetes and its complications. They not only address the complex pathological mechanisms that current drugs cannot solve but also have the potential to slow or even reverse the progression of complications (16). The ability to extract EVs from a patient’s own cells also offers great potential for personalized treatment, providing new ideas and insights for future long-term personalized precision therapies (16). Therefore, in-depth exploration of the mechanisms of EVs in diabetes and their clinical application prospects is of great significance. In the future, through in-depth research on the role of EVs in diabetes and its complications and the continuous exploration of emerging delivery materials, we may discover new therapeutic targets, opening new directions for personalized treatment and drug development.

**Table 1:** miRNA cargo in EVs, associated diseases, effects/targets, and sources.

EV Cargo	Associated Disease	Function and/or Key Targets	EV Source	Reference
miRNA-19b-3p	Type 2 Diabetes	Promotes activation of M1 macrophages and is associated with the progression of kidney injury	Renal tubular epithelial cells	(53)
miR-212-5p	Type 2 Diabetes	Inhibits Sirtuin 2 and downstream Akt/β-catenin pathways, leading to insulin secretion dysfunction	M1 macrophages residing in human islets	(54)
miR-210	Obesity	Inhibits NDUFA4, impairing glucose uptake and mitochondrial activity in 3T3-L1 adipocytes, leading to weight gain	Macrophages from high-fat diet mice	(55)
miR-620	Type 2 Diabetes	Enhances insulin sensitivity in WAT	M2 macrophages in Dicer knockout mice	(56)
miR-3075	Type 2 Diabetes	Compensates for insulin resistance caused by obesity, associated with Akt phosphorylation	Hepatocytes from HFD mice	(57)
lncRNA-p3134	Type 2 Diabetes	Upregulation promotes insulin secretion. Activates PI3K/Akt/mTOR pathway	Mouse islet cells	(51)
miR-128-1	Obesity, Type 2 Diabetes	Upregulation reduces insulin sensitivity, promotes fat accumulation and weight gain	Unknown	(58)
miRNA-126	Type 2 Diabetes	Inhibits phosphatase and PTEN, induces angiogenesis, promotes healing of diabetic foot ulcers	Human bone marrow mesenchymal cells	(59)
miR-let7b/c,miR-126,132	Type 2 Diabetes	Improves penile angiogenesis and downregulates penile fibrosis levels	ADSC/urine-derived stem cells	(60)
miR-486	Type 2 Diabetes	Downregulates SMAD1/mTOR, improves symptoms of diabetic nephropathy	ADSC	(61)
miR-19/103/29/15-b/143	Obesity, Type 2 Diabetes	Upregulation promotes obesity, exacerbates insulin resistance	Human serum	(58)
miR-122、miR-27a 、miR-27b-3p	Obesity	Induces impaired glucose tolerance	Serum from high-fat diet mice	(62)
miR-802	Obesity	Upregulation targets HNF1B, inhibits phosphorylation of IRS proteins, induces insulin resistance	Liver of obese mice	(63)
miR-15a	Type 2 Diabetes	Targets Akt3/PI3 signaling pathway; upregulation exacerbates oxidative stress and apoptosis in retinal cells	Human serum	(64)
miR-15b-5p	Type 2 Diabetes	Induce mesangial cell apoptosis.	Unknown	(65)
MicroRNA-15a	Type 2 Diabetes	Overexpression increases β-cell apoptosis by inhibiting Bcl-2, exacerbating insulin resistance and T2DM progression	Unknown	(66)
miR-1249	Type 2 Diabetes	Improves insulin resistance in hepatocytes by reducing NF-κB activity	Mouse NK cells	(67)
MiR-486-3p	Type 2 Diabetes	Regulates TLR4/NF-κB; upregulation inhibits oxidative stress, inflammation, and apoptosis, delaying diabetic retinopathy	Rats	(68)
MicroRNA let-7c-5p	Type 2 Diabetes	Regulates MAPK, TGF-β signaling, associated with kidney fibrosis in diabetes, potential diagnostic/therapeutic target	Unknown	(69)
IncRNA H19	Type 2 Diabetes	Competes with miR-152-3p, upregulation promotes PTEN, reduces inflammation, and decreases fibroblast apoptosis	Unknown	(70)
MiR-26a	Obesity, Type 2 Diabetes	Induces AKT phosphorylation, improves insulin resistance	Rats fed a high-fat diet	(48)
IncRNA SNHG7	Type 2 Diabetes	Regulates miR-34a-5p, inhibits EndMT and HRMEC, upregulation slows DR progression	Unknown	(71)
microRNA-486-3p	Type 2 Diabetes	Targets TLR4/NF-κB axis, delays diabetic retinopathy progression	Bone marrow mesenchymal stem cells	(68)
microRNA-16-5p	Type 2 Diabetes	Protects podocytes, delays diabetic nephropathy progression	Human urine-derived	(72)
hsa_circ_0000907 hsa_circ_0057362	Type 2 Diabetes	Biomarkers for early diagnosis of diabetic foot ulcers	Human serum	(73)
mmu_circ_0000250	Type 2 Diabetes	Regulates miR-128/SIRT, promotes autophagy, upregulation promotes wound healing in diabetes	Rats	(74)
circEhmt1	Type 2 Diabetes	Upregulates transcription factor NFIA, inhibits angiogenesis, reduces apoptosis, delays diabetic retinopathy progression	Unknown	(75)
circ_DLGAP4	Type 2 Diabetes	Acts as a sponge for miR-143, targets ERBB3/NF-κB/MMP-2, promotes diabetic nephropathy progression	Unknown	(76)
circ_0005015	Type 2 Diabetes	Promotes upregulation of MMP-2, STAT3, promotes DR progression, biomarker for early diagnosis of DR	Unknown	(77)
MiR-222	Type 2 Diabetes	Activate STAT5 protein expression to promote retinal recovery in diabetic retinopathy (DR).	Unknown	(78)

## 2 EVs and diabetes

### 2.1 Potential mechanistic links between EVs and type 2 diabetes

The ESCRT complex and its associated components, along with SNARE proteins, are involved in the formation of extracellular vesicles (EVs) and influence EV-mediated inflammation and oxidative stress, neutrophil lipophagy, lipogenesis, and insulin-stimulated glucose uptake (17–19). For example, SNARE proteins regulate the release of EVs by forming complexes, unwinding structures, membrane fusion, and NSF-mediated complex dissociation, participating in insulin and GLP-1 secretion, as well as GLUT4-mediated glucose uptake, maintaining glucose homeostasis (20). The levels of SNARE protein components (such as SNAP-23 and VAMP2) have been observed to decrease in the islets of diabetic patients, which is closely related to insulin secretion defects, insulin resistance, and impairments involving GLUT4 translocation in these patients (20). MLKL and HSP20 have been shown to interact with ESCRT components to co-regulate EV formation (21–23). Under diabetic conditions, MLKL is upregulated in various tissues. In addition to its pro-inflammatory and cell necroptosis effects, MLKL can independently regulate insulin sensitivity in diabetic mice (24). Meanwhile, HSP20 is downregulated in type 2 diabetes (T2D), and its reduction is considered a major driver of diabetes-related organ damage, while its high expression is associated with improvements in diabetic cardiomyopathy (25).

The plasma membrane undergoes inward budding to form early endosomes, which mature into late endosomes. As this process occurs, intraluminal vesicles (ILVs) are formed through inward budding within the endosome. This process is regulated by proteins such as the endosomal sorting complexes required for transport (ESCRT) system. Ubiquitinated proteins tag specific proteins, facilitating the recognition of late endosomes by the endosomal system, which further develops into multivesicular bodies (MVBs). MVBs either fuse with lysosomes, where they are degraded via the chaperone-mediated autophagy (CMA) pathway with the involvement of Hsc70 (a heat shock protein), or they fuse with the plasma membrane, releasing the ILVs into the extracellular space as EVs. These EVs typically range in size from 30 to 100 nm and contain proteins, lipids, and nucleic acids such as RNA and DNA. Once released, EVs can interact with recipient cells, influencing various biological processes such as immune responses, metabolic regulation, and cell signaling.

### 2.2 EVs and their contents (proteins, RNA) in type 2 diabetes

Diabetic conditions can affect the protein cargo composition of extracellular vesicles (EVs) (26). A longitudinal cohort study (27) indicated that, in a diabetic state, EVs in plasma contain higher levels of inflammation-related proteins (such as Vascular Endothelial Growth Factor A), which promote the formation and migration of endothelial cell pseudopodia, induce vascular dysfunction, and are significantly associated with β-cell function and insulin resistance. Furthermore, animal experiments (28) confirmed that EVs rich in arginase 1 in the serum of diabetic mice could induce vascular lesions in non-diabetic mice by altering the bioavailability of NO, and this harmful effect was significantly reduced after silencing. Previous studies have shown that caveolin-1, a structural protein on the endothelial cell membrane, regulates NO signaling, and NO is closely linked to the regulation of vascular junctional proteins. In its inactive state, Cav-1 directly binds to eNOS through the Scaffolding Domain, inhibiting eNOS activity, preventing its conformational change, and reducing the binding of calmodulin to eNOS, thereby decreasing NO synthesis and bioavailability, impairing vascular function (29). Animal experiments have shown that miR-195 can downregulate eNOS expression by inhibiting SIRT1 (an NAD-dependent deacetylase), affecting transcription factors (such as p53 and NF-κB) to induce metabolic disorders, inflammation, and oxidative stress (30, 31). Based on all previously mentioned points, we propose the following interesting hypothesis to establish the diabetes EVs—Cav-1—NO axis: Diabetic EVs may regulate the expression level of Cav-1 by carrying specific microRNAs, leading to dysregulated eNOS activity and NO bioavailability. The inflammatory response triggered by the molecular cargo of diabetic EVs could even exacerbate the Cav-1 and eNOS interaction disorder through oxidative stress pathways, promoting the development of vascular lesions. Although there is currently a lack of more detailed evidence, this intriguing chain hypothesis points to potential future research directions.

Caveolin-1, CD82, and post-translational modifications (PTMs) play crucial roles in the development of diabetes (32–34). Proteins in EVs are involved in regulating tissue responses to systemic nutritional changes. The endothelial-adipocyte extracellular vesicle axis exists in humans and is controlled by metabolic status (34, 35). Adjacent endothelial cells (ECs) transfer cav1 (caveolin-1) to adipocytes via EVs, with adipocytes interacting with ECs by releasing EVs, creating a reciprocal motion (34, 35). EVs derived from adipose tissue (AT) contain proteins that can regulate cell signaling pathways. This mechanism is modulated by physiological factors such as fasting/refeeding and obesity (34, 35). Under high-glucose conditions, activation of Akt and RhoA requires phosphorylation of caveolin-1 in mesangial cells. Akt and RhoA are associated with insulin resistance and glucose metabolism abnormalities in type 2 diabetes (34). Hypoxia-induced phosphorylation of caveolin-1 promotes the increased release of EVs and sorting of miRNA cargo, which may affect glucose metabolism and insulin signaling (36). Under lipotoxic conditions, CD63 mediates insulin degradation in β-cells, promoting the development of type 2 diabetes (25), while the upregulation of CD63 may be associated with diabetes-related atherosclerosis due to platelet activation (37). Moreover, studies have shown that circulating EVs in the plasma of obese/insulin-resistant patients contain more inflammation and insulin resistance-related proteins (SDCB1, TGFB1). After exercise, EVs showed significant increases in insulin sensitivity-related proteins (PKA, PLC) and oxidative metabolism-related proteins (PRDX1/2, G6PD2, SOD2) (38).

Increasing evidence suggests that miRNAs can bind to EVs and be secreted into the extracellular space, where they are protected from degradation and can circulate stably in the bloodstream (39). Therefore, miRNAs are key carriers of EVs. Circulating EVs carrying specific miRNAs regulate interactions between muscle, fat, immune cells, and stromal cells, influencing the development of type 2 diabetes (39).

MiRNAs intervene in the expression of insulin genes and insulin secretion through various pathways. miRNAs (such as miR-130 and miR-152) regulate insulin expression and secretion by targeting key catalytic enzymes in β-cell ATP generation and glucose metabolism pathways (39). miRNAs broadly interfere with the transcription of insulin genes, such as miR-802 and miR-124a, which inhibit insulin gene transcription by targeting NEUROD1, while miR-30d/26/186 promote insulin gene expression by acting on transcription factors that inhibit insulin transcription (40, 41).

Insulin granules are released through calcium-dependent exocytosis; however, current research evidence suggests that miRNAs appear to play a minimal role in regulating this process. Instead, miRNAs primarily play an important role in reducing the expression of various exocytosis-related genes (e.g., SNARE complex, VAMP2) (42). Under stimulation signals (e.g., calcium influx caused by hyperglycemia), insulin storage vesicles approach the pancreatic β-cell membrane. Syntaxin and SNAP-25 are located on the cell membrane, while VAMP is located on the vesicle membrane. These SNARE proteins form a SNARE complex through mutual interactions, allowing tight contact between the vesicle and the cell membrane, triggering membrane fusion, and ultimately leading to the release of insulin from the vesicle into the extracellular space (43). Animal studies have confirmed that miR-375 regulates the remodeling of the cell protein cytoskeleton and the fusion of secretory vesicles with the cell membrane by targeting myotrophin (a muscle trophic protein) and actin (F-actin), promoting insulin secretion. This process is mainly achieved by reducing the expression of exocytosis-related genes (43).

In the human body, GLP-1 binds to its receptor (GLP-1R), activating G protein-coupled receptor (GPCR) signaling pathways, which in turn activate adenylate cyclase, increasing intracellular cAMP levels, and activating downstream signaling molecules such as protein kinase A (PKA) and the SNARE complex. These complexes directly participate in the fusion and release of insulin vesicles. It has been confirmed that the 3′UTR of GLP-1R is a direct target of miR-204 (44). miR-9 and miR-29 participate in the negative regulation of insulin secretion mediated by granule-associated protein by targeting the transcription factor Onecut2 (45, 46), while miR-24 and miR-96 directly regulate insulin vesicle exocytosis by affecting exocytosis proteins such as SNAP and rab3a (47).

T2DM is a metabolic disease closely related to chronic low-grade inflammation, with macrophages playing a crucial role in this inflammatory response, and miRNAs are closely associated with inflammation. The lncRNA MALAT1 secreted by macrophages cultured under high glucose concentrations may be related to the progression and inflammatory state of T2DM (48). Exosomal lncRNA MALAT1 acts as a “sponge” for miR-150-5p, weakening its function on downstream target genes by binding and inhibiting miR-150-5p, thus affecting the expression of related proteins (e.g., resistin), which may promote the progression of diabetes and its complications through inflammation-mediated mechanisms (48).

Circulating EVs transport a range of miRNAs, such as miR-122 and miR-27, which target PPARα, inducing liver insulin resistance, mild inflammation, and lipid abnormalities (49, 50). Circulating EVs carrying lncRNA-p3134 levels are higher in T2DM patients than in non-T2DM patients and are correlated with fasting glucose and HOMA-β levels (51). Studies have found that lncRNA-p3134 positively regulates glucose-stimulated insulin secretion by promoting the expression of key regulatory factors (Pdx-1, MafA, GLUT2, Tcf712) in β-cells (51). Plasma miR-130a, which targets the AKT-GLUT4 pathway, improves glucose uptake and utilization (37), while lymphocyte-derived EVs rich in miR-155 and miR-142-5p can promote β-cell dysfunction in the pancreas (52). 

### 2.3 Functions of EVs from different tissue sources in type 2 diabetes

#### 2.3.1 Adipocytes

Extracellular vesicles (EVs) from adipose tissue can regulate metabolic homeostasis (26, 79). In EVs derived from the adipose tissue of obese individuals, the expression of miRNA-222 is upregulated, inhibiting the translocation of GLUT4 to the cell membrane, thereby affecting glucose uptake by cells and leading to insulin resistance (80). The miRNA-222 contained in EVs may also trigger lipid metabolism disorders, and its content in EVs is positively correlated with the degree of obesity (80, 81). Additionally, EVs from adipose tissue macrophages transfer miR-155 to insulin receptor cells, targeting PPARγ, impairing insulin signaling, and promoting obesity-induced insulin resistance (81). Compared to healthy individuals, obese individuals with cardiovascular diseases release more EVs from adipose tissue, which are characterized by elevated levels of cystatin C and decreased levels of CD14. Cystatin C is considered an independent risk factor for cardiovascular disease, especially among diabetic patients, potentially involving mechanisms such as deteriorating renal function and atherosclerosis (82). The levels of CD14 in circulating EVs have been observed to correlate positively with leptin (82). While leptin regulates fatty acid release and utilization in adipose tissue, improving glucose uptake and utilization in peripheral tissues and regulating insulin sensitivity, the direct relationship between CD14 and diabetes progression requires further research and evaluation (82). EVs in the adipose tissue of patients can mediate insulin resistance and inflammation, inducing diseases such as non-alcoholic fatty liver disease (NAFLD). Studies have shown that EVs from subcutaneous and omental adipocytes can influence insulin signaling in the liver (82, 83). Moreover, these EVs can regulate the expression levels of gluconeogenic genes such as PEPCK, and the number of EVs from omental adipose tissue has been positively correlated with the degree of liver dysfunction (82, 84). Another study showed that EVs from visceral fat can act on HepG2 cells in the liver, upregulating the expression of TIMP-1 and downregulating MMP-7, regulating extracellular matrix deposition and changes, and inducing liver fibrosis (85). EVs from the adipose tissue of obese individuals induce TGF-β pathway imbalance in HHSteC and HepG2 cells by upregulating TIMP-1 and integrin αvβ-8, a phenomenon not observed in lean individuals’ EVs (85). Adipocyte-derived EVs from obese individuals can induce lipid droplet deposition by delivering neutral fatty acids and trigger endoplasmic reticulum stress and hepatic steatosis through the delivery of lipogenic enzymes and miRNAs (86, 87).

#### 2.3.2 Hepatocytes

EVs released by hepatocytes under lipotoxic conditions are closely associated with liver inflammation and fibrosis. Hepatocytes release pro-inflammatory EVs, such as those containing CXCL10 protein, S1P, TRAIL, integrin β1, and ceramides, which induce chemotaxis of Kupffer cells, activating pro-inflammatory pathways like NLRP3 and ASC, leading to liver inflammation and fibrosis (82, 88–91). The release of hepatocyte EVs is partially regulated by endoplasmic reticulum stress, such as the accumulation of ceramide metabolites like S61P, which further triggers inflammation (92). These pro-inflammatory EVs can also be absorbed by macrophages and hematopoietic stem cells, worsening liver conditions (92). Some important miRNAs, such as miR-7, let-5e-31p, miR-5-210p, and miR-3-130p, when delivered to adipocytes via hepatocyte EVs, may induce adipocyte remodeling, increasing the expression of lipogenic genes and promoting obesity (91, 92).

#### 2.3.3 Islet cells

Under lipotoxic conditions, β-cells experience cholesterol metabolism disorders. In this state, the overexpression of NF-κB and COX-2 may lead to inflammation of the islet microenvironment, while the downregulation of PDX-1 affects insulin secretion capacity (93). High LDL levels affect the cargo sorting of islet cell EVs, inducing insulin resistance by downregulating the mTOR/p70S6Kα pathway (93). Studies have shown that exosomes derived from the pancreas can transfer Mut-Reg1cp to peripheral tissues, promoting insulin resistance by inhibiting AdipoR1 translation and adiponectin signaling (94). In β-cell EVs, the downregulation of miR-26a and upregulation of miR-16/29/155 lead to insulin resistance in the liver and adipose tissues, and monocytes are transformed into inflammatory macrophages. EVs in the plasma of obese individuals may influence β-cell proliferation by regulating the expression of the CD74 gene mediated by miR-7218-5p (95). In insulin resistance, exosomes isolated from the skeletal muscle of obese individuals can bind to Ptch1 3’-UTR in β-cells, downregulating Ptch1 and promoting islet proliferation, with miR-16 playing a key role (96, 97).

### 2.4 Mechanisms of EV-mediated metabolic dysregulation in diabetes

#### 2.4.1 EVs in obesity and insulin resistance

Functional pro-insulin proteins in EVs derived from adipocytes of obese/insulin-resistant individuals have been shown to act as messengers in glucose metabolism regulation, transferring to β-cells, and promoting GPCR/cAMP/PKA signaling through kinase phosphorylation, stimulating insulin secretion. Through this mechanism, β-cells detect insulin resistance in adipose tissue and, if necessary, increase insulin secretion (98). Clinically, the RNA components of EVs derived from endothelial cells, platelets, and monocytes in the plasma of some obese individuals change, which may be closely related to abnormal leptin levels. Abnormal leptin levels lead to AMPK inactivation, affecting insulin signaling. Obesity also leads to the accumulation of pro-inflammatory macrophages in adipose tissue, which secrete pro-inflammatory EVs containing RNA, inhibiting insulin sensitivity in cells (99). Sonic hedgehog (Shh) in exosomes derived from insulin-resistant adipocytes (IRADEs) is a key regulator of M1 macrophage polarization, mediated by the Ptch/PI3K signaling cascade (99). Macrophages treated with IRADE-derived EVs promote insulin resistance in adipose tissue by reducing the expression of insulin receptor substrate-1 (IRS-1) and hormone-sensitive lipase (HSL) (100). EVs from visceral and subcutaneous adipose tissue can target cells marked by FABP4 and CD14, regulating the gene expression of TGF-β and Wnt/β-catenin, impacting insulin signaling and endothelial cell migration processes (101). EVs from the adipose tissue of obese individuals contain miR-141-3p and various adipokines, such as MCP-1, MIF, and IL-6, all of which are involved in regulating insulin signaling (102).

#### 2.4.2 EVs and β-cell apoptosis

##### 2.4.2.1 miRNAs in EVs, inflammation, and β-cell damage

In the early stages of diabetes, certain miRNAs enriched in EVs target key genes that protect β-cell function (103). High glucose levels and inflammation regulate this pattern, leading to the enrichment of specific miRNAs in EVs, contributing to β-cell dysfunction. Experiments show that co-administration of inflammatory factors, such as IL-1β, TNF-α, and INF-γ, causes β-cell damage. In the pancreatic tissue of β-cell-damaged mice, the expression levels of miR-375-3p in EVs released from the pancreas changed significantly (104). Under inflammatory conditions, the miRNA profile of EVs released by MIN6B1 cells (capable of insulin secretion) extracted from mouse embryonic islets also showed significant changes, with miRNAs like miR-146b, miR-290a-3p, miR-195, and miR-497, which are involved in β-cell apoptosis, significantly increasing (103). The increase in monocyte chemoattractant protein 1 and interleukin 27 in islet cell vesicles under inflammatory conditions also induces β-cell damage. Exosomes from fatty liver cells promote β-cell apoptosis by targeting insulin substrate receptor 2 via miRNA-126a-3p, inducing diabetes (105). In contrast, EVs from mesenchymal stem cells overexpressing HIF-1α alleviate hypoxia-induced β-cell apoptosis and senescence through protective autophagy mediated by YTHDF1 (106).

##### 2.4.2.2 EV-mediated crosstalk between β-cells and other tissue cells

Crosstalk refers to the process by which cells influence and communicate with each other through direct contact or the release of signaling molecules. This can involve the release of chemical signals by one cell, affecting the physiological state of nearby cells, or direct cell contact transmitting information. In the context of type 2 diabetes (T2DM), EV-mediated crosstalk involves interactions between β-cells and mesenchymal stem cells, skeletal muscle cells, and adipocytes.

###### 2.4.2.2.1 Crosstalk between β-cells and mesenchymal stem cells

Higher levels of insulin transcripts and C-peptide have been observed in mesenchymal stem cells from T2D patients, but they do not significantly contribute to β-cell insulin secretion in the body (107). Extracellular vesicles (EVs) from mesenchymal stem cells of healthy donors may alleviate β-cell damage caused by autoimmune processes, inflammation, and oxidative stress (108–111). These stem cells can be isolated from human umbilical cord blood, umbilical cords, and adipose tissue (106, 108, 109). In T2DM rodent models, EVs from human umbilical cord mesenchymal stem cells can restore phosphorylation of protein kinase B and IRS, upregulate glucose transporter 4 (GLUT4) expression, promote glycogen synthesis in the liver, maintain glucose metabolism homeostasis, and reverse streptozotocin-induced β-cell apoptosis (112). In porcine islets, EVs from mesenchymal stem cells can prevent β-cell damage caused by hypoxia and oxidative stress after transplantation (109). In β-cells cultured under hypoxic conditions, apoptosis rates increased. However, miRNA-15 in EVs from stem cells was found to protect cells from apoptosis and downregulate endoplasmic reticulum stress (113). Additionally, by transporting proteins in the AKT pathway, exosomes from mesenchymal stem cells inhibit nuclear factor erythroid 2–related factor 2 (Nrf2)-mediated ferroptosis in β-cells, reducing β-cell apoptosis. After polyethylene glycol modification, these exosomes can target islet cells more efficiently, enhancing their protective effects (114, 115).

###### 2.4.2.2.2 Crosstalk between β-cells and muscle cells

Regular exercise has been shown to reduce the incidence of T2D, and the crosstalk between skeletal muscle and β-cells is integral to this effect. EVs released from skeletal muscle after exercise have positive effects on the pancreas (104, 116, 117). Several studies have confirmed the crosstalk between β-cells and muscle cells. Exosomes from palmitate-induced insulin-resistant skeletal muscle in mice were observed to be absorbed by mouse islets or MIN6B1 cells (96). In another study, researchers extracted EVs from the skeletal muscle of palmitate-fed mice, injected them into the tibialis anterior muscle, and tracked them using fluorescent labeling technology. They found that these skeletal muscle-derived EVs accumulated more in the pancreas and muscle, with less accumulation in the liver and spleen (96). *In vitro* experiments showed that skeletal muscle-derived EVs could transfer their cargo to MIN6B1 and 3T3-L1 cells, further validating this crosstalk mechanism (96, 104). EVs extracted from the skeletal muscle of palmitate-fed mice were found to contain higher levels of miR-16, which downregulates the key gene Pitch, delaying T2D progression (96, 104). miR-20b-5p was highly expressed in human myotube cell lines and is involved in glucose metabolism homeostasis (118). This miRNA was also abundant in EVs from the serum of T2D patients (118). When miR-20b-5p levels in skeletal muscle increased, researchers found enhanced glucose metabolism in muscle cells and reduced inflammation, but its overexpression impaired insulin signal transduction (118). Furthermore, EVs released from skeletal muscle after exercise contained various bioactive substances (e.g., oxidative metabolism-related proteins CAT, G6PD2, SOD2, and insulin signaling proteins like PKA), which jointly regulate glucose metabolism in β-cells and other tissue cells (119).

###### 2.4.2.2.3 Crosstalk between β-cells and adipocytes

Adipose tissue is considered a complex and dynamic endocrine organ capable of crosstalk with β-cells through the release of EVs. Literature on β-cell and adipocyte crosstalk is limited to brief summaries. Studies suggest that EVs from healthy human adipocytes can promote insulin secretion under glucolipotoxic conditions in islet cells, maintaining the normal physiological function of β-cells (115). However, EVs from inflamed adipose tissue can lead to β-cell apoptosis and dysfunction, possibly due to the upregulation of miR-155/146 (115). Inflammatory factors in adipocyte EVs regulate targets like GSK-3β and ERK1/2, interfere with JNK phosphorylation, and alter CHOP mRNA levels, impacting β-cell survival and proliferation (115). EVs from visceral fat in obese individuals were observed to have upregulated levels of miR-27a-5p, which acts on L-type Ca2+ channel subtype CaV1.2, inducing β-cell dysfunction and damage. Depletion of this miRNA cargo improved glucose tolerance and insulin secretion in experimental rats (120). In another study, EVs from 3T3-L1 adipocytes treated with inflammatory cytokines (CKs) were co-incubated with EndoC-βH3 β-cells and human islets, and the results showed that EVs from inflamed adipose tissue had a negative impact on β-cell survival and insulin secretion. This confirmed the crucial role of inflammation in the β-cell–adipocyte crosstalk (115).

EVs carry different miRNAs that regulate insulin sensitivity, lipid metabolism, and glucose uptake in adipose tissue, liver, pancreas, muscle, and intestine.

### 2.5 EVs and inflammation

Macrophages are an essential part of the immune system and play a crucial role in the development of diabetes. Macrophages can differentiate into different phenotypes, including M1 (pro-inflammatory) and M2 (anti-inflammatory) types. Extracellular vesicles (EVs) released by M1 macrophages contain high levels of pro-inflammatory factors such as TNF-α, IL-6, and IL-1β, which trigger inflammation, disrupt insulin signaling pathways, and induce insulin resistance in adipose tissue. In contrast, M2 macrophages can suppress inflammation and improve insulin sensitivity (121). The upregulation of tyrosine hydroxylase in M2 anti-inflammatory macrophages promotes the release of catecholamines, which in turn promotes the proliferation of ADSCs (adipose-derived stem cells), helping to reduce obesity, alleviate metabolic disorders, and enhance immune homeostasis (121). The formation of M1 macrophages is closely related to the release of more EVs from inflamed adipose tissue, specifically involving retinol-binding protein 4 in EVs acting on the interleukin-1 receptor domain, interfering with the TLR4/TRIF pathway (121).

In the islets, EVs released by β-cells containing miR-29 mediate crosstalk between macrophages and β-cells by acting on TRAF3. Upregulation of this miRNA promotes the recruitment of monocytes and inflammatory macrophages, leading to insulin resistance and systemic glucose metabolism disorders (112). Another study observed lower levels of miRNA-324-5p in EVs released by vascular smooth muscle cells under high-glucose conditions. miRNA-324-5p targets CPT1A and upregulates its expression, inhibiting the release of inflammatory factors from vascular smooth muscle cells and improving vascular inflammation (122).

Additionally, EVs released by the gut microbiota can alter the insulin sensitivity of insulin-target cells, recruit inflammatory cells, and induce metabolic dysfunction. Dysbiosis of the gut microbiota in patients with metabolic diseases is associated with increased gut permeability and the translocation of pathogen-associated molecular patterns into the bloodstream, triggering metabolic endotoxemia, leading to mild systemic inflammation and insulin resistance in metabolic syndrome (123, 124). In a randomized crossover trial involving an oat-rich diet (OTA), participants experienced reduced levels of EVs released by macrophages, monocytes, and platelets, along with decreased levels of fibrin(+) and P-selectin(+) PMP in their serum after 8 weeks on the diet. These outcome measures are closely associated with systemic inflammation and cardiovascular risk in diabetes (125).

## 3 Diabetes complications

### 3.1 Diabetic nephropathy

Diabetic nephropathy (DN) refers to the pathological process of kidney damage caused by prolonged hyperglycemia, characterized by a gradual decline in glomerular filtration rate, proteinuria, hypertension, and renal dysfunction. The related mechanisms include hyperglycemia-induced damage to the glomerular filtration membrane, extracellular matrix proliferation, inflammation, oxidative stress, and others. Increasing evidence suggests a correlation between EVs and DN (61, 126).

The pathogenesis of DN involves EV-mediated intercellular communication. In a hyperglycemic environment, EVs participate in kidney intercellular communication, leading to epithelial-mesenchymal transition, apoptosis, inflammation, and fibrosis, contributing to kidney injury (127, 128). Studies have confirmed that renal tubular epithelial cells exposed to lipotoxicity release EVs that promote macrophage transition to an inflammatory phenotype (mediated by LRG1 and TGFβR1). In turn, these inflammatory EVs promote apoptosis of renal tubular epithelial cells (via TRAIL acting on DR5), and this process involves a negative feedback communication mechanism. In addition, miR-92a-1-5p derived from proximal tubular exosomes under glucolipotoxicity acts on mesangial cells and can induce their apoptosis (126, 129). In diabetes, EVs released by glomerular capillary endothelial cells can deliver mRNA to mesangial cells, causing mesangial cell proliferation and kidney fibrosis (130–132). Podocyte-derived EVs under high-glucose conditions transport miR-221-3p, inducing dedifferentiation of renal tubular epithelial cells through the Wnt/β-catenin pathway (128). Chronic inflammatory stimulation contributes to DN’s progression and persistent damage, and EVs are also involved in this process. Research has shown that EVs from macrophages treated with high glucose upregulate miR-21-5p levels, leading to podocyte apoptosis (133). Macrophage-derived EVs induce monocyte differentiation into inflammatory macrophages by regulating the NF-κB p65 pathway, further releasing more inflammatory factors that lead to kidney damage and inflammation (130, 133). Through the TGF-β1/Smad3 signaling pathway, mesangial cell activation is triggered, resulting in excessive extracellular matrix deposition and increased release of inflammatory factors like IL-1β, further causing mesangial expansion and kidney fibrosis (15, 134). Adipose-derived stem cell exosomes regulate the Nrf2/Keap1 pathway in diabetic nephropathy by targeting FAM129B, alleviating oxidative stress and inflammation in high glucose-induced podocyte injury (135).

EVs containing miR-16-5p can inhibit VEGFA and podocyte apoptosis, restoring kidney function in DN (136). Exosomes secreted by MSCs overexpressing miR-15-5p alleviate kidney damage in diabetic rats by promoting autophagy via the mTOR pathway (137). Similar effects have been observed with exosomes extracted from adipose-derived stem cells (ADSCs) (137).

### 3.2 Diabetic retinopathy

DR is one of the most common microvascular complications of diabetes. The pathophysiology of DR is highly complex, involving changes in neuroglial cells and the structure and function of microvessels, potentially related to increased polyol pathway flux, the generation of advanced glycation end products (AGEs), oxidative stress, and protein kinase C (PKC) activation, although many hypotheses remain unverified in human studies (138). DR may also be closely associated with chronic inflammation, increased expression of vasoactive factors, and cytokines (139).

EVs from different cell types, including endothelial cells, platelets, monocytes, and retinal pigment epithelial cells, participate in the onset and progression of DR (140–143). miRNAs in EVs play a key role, for instance, miR-150-5p/miR-21-3p may cause pericyte loss and induce retinal microvascular abnormalities (144). Research has found that decreased expression of microRNA-222 in retinal tissue is associated with widespread hemorrhage and severe retinal damage in diabetes. microRNA components in small EVs from MSCs participate in diabetic retinopathy, where the transfer of microRNA-222 can enhance retinal regeneration, and microRNA-126 inhibits retinal inflammation in diabetic patients by downregulating the HMGB1 signaling pathway (145). Additionally, regulatory steps in the EV generation process lead to the significant accumulation of MMP-14 in neovascular membrane vesicles, which may promote neovascularization (146). EVs contribute to thrombosis in DR, particularly by increasing pro-coagulant EVs (147). In diabetic animal models and the eyes of diabetic patients, elevated levels of pro-coagulant EVs carrying prothrombin have been observed, along with a significant increase in TF-EVs, which is consistent with elevated markers of coagulation activation (148). Furthermore, pro-coagulant EVs from endothelial cells, platelets, and monocytes in diabetic patients are significantly increased. Platelet-derived EVs tend to attach to the vascular endothelium of diabetic patients, potentially leading to occlusion of damaged retinal capillaries (149, 150).

### 3.3 EVs and cardiovascular complications in diabetes

Cardiovascular complications of diabetes refer to severe cardiovascular system complications caused by long-term diabetes, with coronary heart disease being a typical example. EVs are involved in disease progression, including interference with endothelial function, platelet activation, and angiogenesis (151). EVs can induce endothelial dysfunction. Diabetes may damage the endothelium, leading to endothelial dysfunction, which can cause vasoconstriction, thrombosis, and inflammation, ultimately increasing the risk of heart disease (151). *In vitro* studies have shown that EVs from obese rats upregulate VCAM-1 expression and increase oxidative stress in endothelial cells (152, 153). EVs are also involved in diabetes-induced myocardial infarction, closely related to the platelet activation process. Researchers compared the EV expression profiles of myocardial infarction patients (including STEMI and NSTEMI). Despite dual antiplatelet therapy, more EVs, mainly from platelets, were observed in the plasma of STEMI patients (154). The plasma TF pro-coagulant activity of STEMI patients was higher and positively correlated with the number of EVs from platelets, monocytes, and TF-carrying EVs (154). Moreover, diabetes affects the abundance of pro-angiogenic and anti-angiogenic miRNAs. Compared to healthy individuals, the abundance of these EV-associated miRNAs is dysregulated in T2DM patients, suggesting a link to vascular complications resulting from impaired angiogenesis in this patient group. The levels of dysregulated miRNAs isolated from circulating EVs in T2DM patients (such as miR-193b-3p, miR-199a-3p, miR-20a-3p, miR-26b-5p) were significantly altered (155). Another study found that the levels of miR-126 and miR-26a isolated from circulating EVs in T2DM patients were significantly reduced compared to non-diabetic patients, and these miRNAs have potential roles in reducing coronary atherosclerosis (156). 

EVs, through carrying miRNAs, regulate the inhibition of insulin signaling pathways (such as GLUT4 and Akt), exacerbating insulin resistance. Low expression of GLUT4 is associated with insulin resistance, dyslipidemia, hyperglycemia, and increased inflammatory responses. This can induce the proliferation, migration, and phenotypic transition of arterial endothelial cells. In the insulin signaling pathway, when insulin binds to its receptor, it first activates IRS (insulin receptor substrate), which then activates PI3K (phosphoinositide 3-kinase), initiating downstream AKT (protein kinase B) signaling. Under diabetic conditions, AKT inactivation leads to reduced release of eNOS (endothelial nitric oxide synthase), inhibiting nitric oxide (NO) production, which results in impaired vasodilation and induces endothelial dysfunction in the arteries. At the same time, abnormal insulin signaling affects phosphorylated myosin light chain kinase (p-MLCK), further impacting vascular smooth muscle cell contraction and endothelial barrier function. Any disruptions in this pathway, such as insulin resistance or weakened IRS or PI3K signaling, can lead to AKT/eNOS dysfunction and reduced NO production, causing inflammation and endothelial dysfunction. Low-density lipoprotein (LDL) promotes the generation of reactive oxygen species (ROS), and excessive ROS production damages endothelial cells, promoting the oxidation of LDL into oxLDL. This exacerbates inflammatory responses and foam cell formation, further accelerating the progression of atherosclerosis, creating a vicious cycle.

### 3.4 Diabetic cardiomyopathy

In patients with diabetic cardiomyopathy, the expression level of miR-320 is significantly increased and participates in the development of diabetic cardiomyopathy by regulating multiple target genes, potentially involving the regulation of biological processes such as apoptosis, inflammation, and fibrosis (157). Dysfunctional adipocyte-derived small extracellular vesicles (sEVs) are enriched with miR-130b-3p, which exacerbates myocardial cell injury in diabetic rats by inhibiting various anti-apoptotic/cardioprotective molecules in cardiomyocytes, specifically involving the regulation of downstream targets AMPKα2, Birc6, and Ucp3, as well as abnormal communication between adipocytes and cardiomyocytes (158). miRNA-195, -125b, -199a, and -124 significantly regulate pathological cardiac hypertrophy and heart failure under diabetic conditions (159). The overexpression of cardiomyocyte extracellular vesicles rich in HSP20/27/70 can exert protective effects on cardiomyocytes *in vitro* under high glucose stress. Among these, extracellular vesicles rich in HSP20 can significantly improve the left ventricular end-diastolic diameter and ejection fraction in diabetic mice, alleviating adverse cardiac remodeling (160). Extracellular vesicles derived from mesenchymal stem cells improve myocardial injury and fibrosis caused by diabetes by inhibiting the TGF-β1/Smad2 signaling pathway (15).

## 4 Prospects and challenges

### 4.1 Future prospects of EVs

Exosomes have significant advantages in minimally invasive diagnostics for diabetic patients. On a technical level, exosomes can be isolated from various body fluids (blood, urine, saliva, etc.), meaning they can be conveniently collected using minimally invasive or non-invasive methods, avoiding the trauma associated with traditional diagnostic approaches (161). The rich biomarkers contained within EVs (such as proteins and miRNAs) can reflect the pathological state of cells, closely linked to specific biological processes of diabetes and its complications, thus providing strong evidence for early diagnosis and progress monitoring of the disease (162, 163). Additionally, the phospholipid bilayer structure of exosomes protects the biomolecules inside from degradation by the external environment (such as proteolytic enzymes), thus increasing the stability of the markers in body fluids, enhancing the flexibility of sample preservation and handling, and aiding in long-term storage and subsequent detection (164). The laboratory techniques related to exosomes are relatively mature, with a wide range of applications in separation, characterization, and analysis techniques, including density gradient ultracentrifugation, immunocapture, transmission electron microscopy, protein/gene chip analysis, and liquid chromatography (LC/MS) (165, 166). At the patient level, there are significant differences in the quantity and composition of EVs between diabetic and non-diabetic patients (167, 168). For instance, a significant increase in EVs derived from monocytes, endothelial cells, and platelets has been observed in the circulation of diabetic patients, which is associated with inflammation cell activation and endothelial cell apoptosis under hyperglycemic conditions. Hyperglycemia often promotes vascular inflammation, increasing the activity of NADPH oxidase released in EVs by endothelial cells (167, 168). Nanoparticle tracking analysis and flow cytometry can be used to quantitatively assess and characterize EVs by tracking the Brownian motion of exosome particles and fluorescence-labeled detection, respectively (169–171). As previously mentioned in this article, variations in the types and quantities of GLP-1, proteins, and miRNAs abundant in EVs often occur before symptoms appear, further underscoring their significance in early diagnosis. The combination of exosomes with emerging materials also holds great application prospects. Studies have shown that loading exosomes into smart hydrogels/nanoparticles, combined with 3D printing technology or LBL self-assembly technology, can effectively promote the healing of diabetic wounds (172). By combining chitosan/silk hydrogel sponges with GMSC-derived exosomes, researchers have found accelerated wound healing in diabetic skin defect rat models treated with exosomes (173). Nanoliposomes, as EV carriers, not only enhance their stability but can also extend blood circulation time and improve targeting by altering their surface structure (such as PEGylation), facilitating effective delivery of EV cargo through cell membrane fusion. In models of diabetic nephropathy, EVs delivered via nanoliposomes significantly improved renal function and reduced inflammatory responses (174).

### 4.2 Shortcomings and potential challenges

As discussed in this article, despite positive research progress in disease diagnosis, prediction, and treatment, urgent issues related to EVs, such as the stability of cell phenotypes, large-scale production methods, and precise targeting delivery of EVs, still need to be addressed (175–177). Currently, suspended cell lines (such as tumor cell lines) are often used in bioreactors (such as large-scale culture tanks) along with appropriate culture media and controlled conditions to achieve higher cell density and EV yield (175). However, various factors such as temperature, pH, dissolved oxygen, culture medium formulation, cytokines, and early or late cell passage can affect the phenotype of cells and their derived EVs (175, 178, 179). Some researchers have developed a culture medium primarily composed of human platelet lysate, targeting EVs derived from human mesenchymal stem cells, achieving stabl and reliable characterization of their protein and RNA components while preserving stem cell differentiation potential (180). Appropriate methods for EV separation are also crucial for clinical diagnosis and treatment (181). Some researchers have effectively isolated extracellular vesicles for component and functional studies using ultrafiltration size exclusion chromatography (UF-SEC) and ultrafiltration techniques (178–180). Phosphate-buffered saline, with an osmotic pressure and pH close to physiological levels, is easy to prepare and cost-effective, making it used by some researchers for EV preservation, though the formation of calcium phosphate during preservation can interfere with subsequent quantitative detection (180).

To minimize adverse secondary reactions of therapeutic exosomes, it is also crucial to explore the organ tropism and biodistribution of therapeutic exosomes in depth. In terms of targeting receptor cells, research has confirmed that the targeting ability of EVs is closely related to their compositional components (proteins, glycoproteins, etc.). Different integrin profiles (such as Tetraspanin profiles) can target EVs to different organs (brain, liver, etc.) (182). Other studies have confirmed that the complex of Tspan8 and integrin α can selectively target EVs to cells in the pancreas. This mechanism enables EVs to enhance their uptake and function in the pancreatic microenvironment by binding to specific receptors on the surface of target cells (183).

Regarding the targeting or internalization of EVs, current challenges mainly involve exploring precise delivery of EVs to action sites while avoiding accumulation of EVs in unintended sites of action. This can be achieved by genetically modifying EVs, adding targeting components (such as nanobodies and antibodies), and developing multifunctional targeting peptides (184). Some studies have found that combining Lamp2b on the surface of EVs with rabies virus glycoprotein peptides can target EVs to brain neurons. Researchers have fused nanobodies to the mucin domain of EVs, enhancing the binding ability of EVs to phosphatidylserine while preserving the integrity of EVs, thereby facilitating their uptake (185). A class of RGD peptides, which bind to integrins expressed on the surface of newly formed blood vessels, can be fixed to the surface of EVs by combining with PEG lipids, allowing precise targeting of αvβ3 cells, which may promote angiogenesis and provide insights for EV treatment of diabetes-related vascular lesions (186). By using a cycloaddition reaction of sulfonyl azides, researchers combined the targeting peptide RGERPPR with EVs, enabling effective passage of EVs across the blood-brain barrier and precisely targeting nerve cells (187). These may inspire and inform future EV treatments for diabetes and its complications.

It is worth noting that the potential safety and efficacy of EVs require further evaluation. Research on pharmacodynamics, biodistribution, pharmacokinetics, as well as unknown immune responses and bioactivities remains limited. There is a severe lack of studies on the optimal dosage for EVs treatment, and we still know very little about the short- or long-term harmful effects caused by improper EV dosage. Furthermore, the transparent sourcing of therapeutic EVs is considered critical. RNA and DNA fragments carried by exosomes from tumor cells or unhealthy cells may have carcinogenic effects, and the non-specific actions of EVs may also affect other types of cells, inducing unknown cellular changes by transferring bioactive molecules. For instance, a study confirmed that MSC-EVs derived from human umbilical cord directly inhibit the expression of PTEN via miR-410, strongly promoting the growth of lung adenocarcinoma cells in tumor-bearing mice, whereas siR-410 could eliminate this effect (188). Similarly, Hedgehog and β-catenin in EVs derived from breast cancer stem cells can enhance the resistance of existing tumor cells to various cancer therapies (189). This also highlights the potential ethical issues in EVs therapy, such as how to ensure transparent traceability of exosome sourcing and how to guarantee patients’ full informed consent in the face of emerging technologies. The possible involvement of gene editing technologies and the unknown long-term effects of EVs therapy also pose ethical challenges. More research is still needed in the future to fully clarify the mechanisms of EVs, mitigate risks, and enable them to contribute more effectively to clinical treatment.
